# Health Economics in the Field of Prosthetics and Orthotics: A Global Perspective

**DOI:** 10.33137/cpoj.v4i2.35298

**Published:** 2021-09-21

**Authors:** A Kannenberg, S Seidinger

**Affiliations:** 1 Otto Bock Healthcare LP, Austin, Texas, USA.; 2 Otto Bock Healthcare GmbH, Vienna, Austria.

**Keywords:** Disability, Health Economics, Prosthetics, Orthotics, Health Technology Assessment, Manufacturer, Reimbursement, Research and Development, Willingness to Pay

## Abstract

The rapid advancement of prosthetic and orthotic (P&O) technology raises the question how the industry can ensure that patients have access to the benefits and providers get paid properly and fairly by healthcare payers. This is a challenge that not only P&O but all areas of health technology face. In many areas of medicine and health products, such as drugs and medical devices, health-technology assessments (HTA) have become a standard procedure in the coverage and reimbursement process. In most countries, P&O is lagging behind that development, although some countries have already formalized HTA for prosthetic and orthotic products and may even use cost-effectiveness analyses to determine pricing and payment amounts. This article gives an overview on the coverage and reimbursement processes in the United States, Canada, Germany, France, Sweden, the United Kingdom, Poland, Japan, and China. This selection reflects the variety and diversity of coverage and reimbursement processes that the P&O industry faces globally. The paper continues with an overview on the necessary research and investment efforts that manufacturers will have to make in the future, and contemplates the likely consequences for the manufacturer community in the market place. Health economics may help support the transition from price-based to value-based coverage and reimbursement but will come at considerable costs to the industry.

## INTRODUCTION

With populations aging and medical progress accelerating, the financial pressure on health care systems around the world is constantly increasing. As the resources that can be spent on health care are limited, policymakers have the challenge to balance spending and health outcomes while preventing possible inequalities in beneficiary access, such as age discrimination of the elderly.

Health technology assessments (HTAs) are performed by synthesizing information and evidence on the clinical, economic, social, and ethical value of health technologies with the objective to inform safe and effective health policies, especially with respect to appropriate coverage and reasonable reimbursement. Health-economic and budget-impact analyses originally started in the early 1990s and have developed into a standard tool to inform decision-making for pharmaceuticals. Analyses have meanwhile expanded to medical devices and clinical procedures using modified methodology that better reflects their specific needs. As medical devices differ considerably from pharmaceuticals, there is still a need to refine the HTA approach to these technologies.^1,2^ While common for medical devices used in hospitals or by physicians, health economics is still in its infancy in the field of prosthetics and orthotics (P&O). Even worse, most P&O products, with notable exceptions, such as microprocessor knees, still lack a solid body of evidence for meaningful clinical benefits that would be required to determine the effectiveness side of a cost-effectiveness equation. There is a considerable amount of biomechanical research that may have even shown promising findings in the gait lab. However, many manufacturers still fall short in conducting meaningful clinical research. For example, while increased toe clearance in the gait lab was shown for prosthetic feet with hydraulic and microprocessor-controlled ankles years ago^3,4^ no study has been published yet to show that these feet reduce falls in the free-living environment. Having come to the conclusion that most manufacturers do not conduct robust and meaningful clinical research,^5–8^ let alone cost-effectiveness studies, health-technology assessment bodies that have reviewed the evidence for some P&O products usually list the deficiencies in the evidence and, therefore, often abstain from compelling coverage recommendations.

Though there are some basic methodologic commonalities of health-economic analyses for all health technologies, a specific challenge in P&O is the relative lack of expertise in the field of disability that would be necessary to develop health-economic models that appropriately represent and reflect the specific needs and outcomes of patients. According to a recent survey, over 80% of health-economic experts said they knew far too little about this area.^9^ So, it is not surprising that currently no specific health-economic model exists that appropriately reflects the conditions and needs of patients with disabilities treated in P&O. That also means that manufacturers have no reliable guidance to satisfy the expectations of HTA bodies. Nevertheless, health-economic evaluations will very likely become an important aspect of future coverage and reimbursement decisions. Therefore, the P&O industry would be well-advised to proactively approach health economics soon to be able to shape its framework rather than to wait for inadequate models to be imposed from the outside.

This paper aims at giving an overview on health-economic requirements for coverage and reimbursement policies in important health care systems around the world. However, regardless of a requirement, cost-effectiveness analyses would also bring the industry in a position to argue for the value of its services and products by putting it in perspective to that of other medical services and products. For example, a health-economic study in the United States found that the incremental cost-effectiveness ratio (ICER) of microprocessor-controlled prosthetic knees was comparable to that of total knee replacements but much better than that of prophylactic cardioverter defibrillator implantations.^10^ Such findings might help influence policies even without a formal requirement for health-economic evaluations.

## CURRENT REIMBURSEMENT SYSTEM CHALLENGES FOR PROSTHETICS AND ORTHOTICS

### Decision-making process for coverage

Currently, in the majority of health care systems worldwide, health-economic studies are not a standardized part of the decision-making process for P&O coverage, issuance of a new billing code, if applicable, or patients' access to treatment.

While public insurance infrastructure and appraisal procedures for pharmaceuticals are generally clearly regulated and medical devices are increasingly approaching these standards, coverage appraisal systems for P&O usually lag way behind and still focus primarily on clinical evidence, if any. One of the reasons is that P&O is only a tiny field in healthcare, making up less than 0.3% of the 2018 overall Medicare budget in the U.S.^11,12^ However, the fact that individual prostheses may cost tens of thousands of dollars creates a psychological barrier with claim reviewers on the insurance side, sparking their wish to see good justification for such a big expenses. Therefore, health-economic analyses may help put the cost and benefits of an advanced prosthesis or orthosis in perspective to those of other medical interventions that are considered standard of care.

## OVERVIEW ON THE ADOPTION OF HEALTH ECONOMIC EVALUATIONS IN THE FIELD OF PROSTHETICS AND ORTHOTICS IN DIFFERENT COUNTRIES

For this paper, countries were selected that have a formal process to enable manufacturers to provide scientific evidence for the assessment of their products and that publish appraisal results.

### NORTH AMERICA

#### UNITED STATES

In 2018, U.S. health care spending totaled $3.6 trillion, amounting to 17.7% of the gross domestic product (GDP) or US$11,200 per capita — almost twice as much as many other high-income countries.^13^ The biggest expenditure categories are hospitals and physicians. Though the amount spent on prescription drugs is growing, the proportion of health care spending for drugs is fairly stable.^14^ Interestingly, public health care expenditures as a share of the GDP (8.3%) are comparable to other large economies. However, public health insurance in the United States (Medicare, Medicaid, Children's Health Insurance Program [CHIP], Veterans Administration [VA]) covers only 34 percent of the population, much less than in countries with universal coverage like Canada and the United Kingdom.^15,16^ That indicates that it costs far more to provide health care coverage in the U.S. system than anywhere else in world. Despite leading the world in costs, however, the United States ranks only twenty-sixth in the world for life expectancy^17^ and also ranks poorly on other indicators of quality.^18^ Thus, there would be good reason to employ health-economic evaluations to improve outcomes and manage health care costs. However, the information on the impact of health-economic analyses on the U.S. health care system is conflicting. On one hand, the Department of Health and Human Services (HHS) in 2016 issued Guidelines for Regulatory Impact Analysis that apply to any regulatory actions that have an annual effect on the economy of US$100 million or more.^19^ On the other hand, Medicare is prohibited by law from using cost-effectiveness in coverage determinations.^20^ That is reflected by the fact that the process for the development and revision of Local Coverage Determinations (LCD) that also applies to prosthetics and orthotics does require the submission of clinical evidence on new products published in peer-reviewed medical journals, but not the submission of any health-economic evaluations of the new technology.^21^

Commercial health care insurances have more discretion with regards to health economics. However, the Affordable Care Act, though encouraging cost-effectiveness and comparative clinical research, also restricts the use of that evidence for determining coverage and reimbursement.^22^ Nevertheless, health-economic analyses of new interventions, drugs and medical devices are quite common in the U.S. However, Medicare and commercial insurances have separate departments and processes to determine coverage and payment amounts.

For coverage, they usually focus on published clinical evidence and the methodological quality of the research conducted, regardless of the price or reimbursement requested. Health-economic analyses are usually not considered for coverage decisions. Only after a new intervention has been found to be effective and worth covering, health-economic analyses may be used to help determine an adequate payment amount by providing the cost-effectiveness perspective that may be compared to that of other interventions already covered. Due to the known challenges to meet the formal criteria of high-quality clinical research in prosthetics and orthotics, such as blinding and randomized parallel groups, manufacturers often fail to even pass the first gate of evaluation of the evidence for clinical benefits and effectiveness. However, if payers even dispute the clinical effectiveness of prosthetic and orthotic devices and deny coverage for lack of evidence thereof, there is no need to analyze cost-effectiveness to guide determination of pricing and reimbursement. That was basically confirmed by the experience that a health-economic evaluation of microprocessor controlled prosthetic knees (MPK) commissioned by the American Orthotic and Prosthetic Association (AOPA) and conducted by the RAND Corporation^10^ was widely ignored by healthcare payers and lawmakers. For community ambulators (K3), the study confirmed that the previous decision to cover MPK was cost-effective, whereas for limited community ambulators (K2), the results were rejected for insufficient clinical evidence as a solid basis for a cost-effectiveness analysis. Given the dire evidence situation for many P&O products, “health economy” is mostly interpreted as cost containment and payment reduction by payers, be it through “least costly alternative” provisions, wide and more inclusive interpretation of billing codes (exclusion of “unbundling” of features and functions), and contracts with ever-dwindling payment rates. These challenges are a clear indication that U.S. healthcare payers have not yet understood the value of P&O care. Another issue, especially for innovative manufacturers, is that payers clear coverage for billing codes rather than specific products. In most cases, coverage of a billing code is approved based on the evidence for one specific product that is usually the predicate device for that code. However, once coverage of that code is approved, followers can take advantage of it with me-too products that do not need evidence for their clinical effectiveness anymore and can, thus, be offered at a lower price.

#### CANADA

Canada spends about 11.1% of its GDP on healthcare, comparable to the level of other high-income countries.^23^ The Canadian healthcare system is predominantly funded publicly with only 30% of funding coming from the private sector. The federal government provides health care funding to the 13 provinces and territories through the Canada Health Transfer and other fiscal transfers.^24^ Provincial and territorial health authorities have the responsibility to meet the basic health service requirements of the Canada Health Act (CHA) that requires coverage of hospital services, physician services, surgical-dental services provided by hospitals, medical practitioners or dentists.^25^

Outside the basic health services mandated by the CHA, provinces and territories have the power to decide what packages of services they will provide. This provincial independence has resulted in a large variation of coverage between provinces.^26–28^ Per capita spending in 2016 for all of Canada was projected to be CAN$6,299, but spending by province ranged from CAN$5,822 in Québec to a high of CAN$7,256 in Newfoundland and Labrador.^23^ These variations in coverage do also and specifically apply to prosthetics, ranging from no formal coverage policies and prosthetic coverage in Newfoundland & Labrador and Prince Edward Island to coverage of up to CAN$17,690 towards select advanced components in Ontario.^28^

Health Technology Assessments (HTA) started in Canada about 25 years ago with the establishment of the Conseil D'Evaluation des Technologies de la Santé (today: Agence des Technologies et des Modes Intervention en Santé [AETMIS]) in Quebec, soon followed by the Canadian Coordinating Office of Health Technology Assessment (today: Canadian Agency for Drugs and Technologies in Health [CADTH]) at the federal level. In addition, many of the larger provinces have their own independent bodies, such as the Ontario Health Technology Advisory committee (OHTAC). However, in the past, economic factors were not nearly as predictive of recommendations by the HTA agencies as clinical reasons and clinical certainty of effect. Also, the health-economic approach has usually been more simplistic, focusing on unit cost comparisons rather than true cost-effectiveness. For example, the odds of a drug being recommended were nine times higher if that drug was cheaper than a comparator drug, regardless of absolute cost-effectiveness.^28^ That may change with increasing financial pressure on the healthcare system in the future. However, a recent study even suggested using different thresholds for cost-effectiveness analyses for each of the 13 provinces and territories based on disability-adjusted life years (DALY) averted and the actual level of health care expenditures in the respective province rather than one national threshold for Canada.^29^ While such an approach might be considered appropriate from the perspective of provincial health authorities, it would make it much more difficult for providers and manufacturers to navigate the Canadian healthcare system.

To our knowledge, only the C-Leg has been subjected to formal reviews of clinical evidence and cost-effectiveness by both the Evidence Based Practice Group of WorkSafe BC in 2003, updated in 2009,^6^ and CADTH in 2009,^7^ both with favorable recommendations. However, not all provinces and territories have adopted them. Similar as the situation in the United States, the most likely reason is the limited methodological quality of most of the prosthetic research that does not provide a solid basis for cost-effectiveness analyses. Thus, the quality of clinical research in prosthetics needs to be improved first.

#### EUROPE

The European Network for HTA (EUnetHTA) enables the European Union to perform one HTA for several or all European countries to reduce research efforts. Both national agencies and manufacturers can initiate an evaluation. The focus of EUnetHTA is on medical devices of classes III and II. However, other technologies might open the door for class I.^30^ The Austrian HTA agency has been chosen by EUnetHTA to prioritize projects for other technologies and has already started a national assessment of exoskeletons and functional electrical stimulation in stroke rehabilitation units in Austria. In general, health economic studies are only considered if published in a peer-reviewed journal. The interaction between HTA bodies and manufacturers is refined by the obligation of the latter to continuously document the medical benefits and safety of their medical products along their life cycle.^31^

However, it must be considered that EUnetHTA's recommendations are not legally binding for EU countries and that the implementation of this network is far from being completed. Therefore, in the next section we present the diversity of the evaluation of health-economic evidence across select European countries.

#### GERMANY

In 2018, Germany spent €390.6 billion (US$460.9 billion) or €4,712 (US$5,560) per capita on health care, equaling 11.7% of the GDP.^32^ The statutory health insurance provides comprehensive medical coverage to 90% of the population, with premiums depending on income.^33^ In principle, people with disabilities are entitled to coverage of state-of-the-art prosthetic and orthotic devices in accordance with the statutory provisions of the Social Code (Sozialgesetzbuch [SGB]; §4 and §47 SGB IX;^34^ §33 and §27 SGB V^)35,36^ as soon as the CE mark is approved in Europe. In addition, the Federal Social Court adjudicated for MPKs that patients are entitled “*to receive aids that compensate the disability and enable equal function for activities of daily living as an able-bodied person*”.^37^

To be listed in the Directory of Medical Aids (“GKV Hilfsmittelverzeichnis”), the clinical benefit of a medical device must be demonstrated. Under certain circumstances, research questions for the listing of new device categories may be negotiated. Usually, the insurances negotiate contracts and reimbursement amounts with the Federal Guild of Prosthetists and Orthotists based on the directory. In principle, the Federal Joint Committee (G-BA) of Physicians and Health Insurances, the highest decision-making body for coverage in the German healthcare system, would have the authority to commission a health-economic evaluation by the HTA body IQWiG (Institute for Quality and Efficiency in Health Care). This has been never done for prosthetics or orthotics thus far.^38^ If a manufacturer conducts a health-economic study in a German context, the IQWiG methodology is to be followed and modeled on a German cohort.^39^ An example is the cost-effectiveness study and budget impact analysis for C-Leg in a German context.^40^ The value of a health-economic evaluation may inform statutory health insurances and help grant extended access for sub-groups of patients which are likely perceived as cost-intensive.

#### FRANCE

The Haute Authorité de Santé (HAS)^41^ has a published, clearly defined appraisal process including instructions for the interaction with manufacturers that is to be followed for drugs, medical devices and P&O equally. This is also noticeable in the assessment expertise of the Commission *nationale d’évaluation des dispositifs médicaux et des technologies de santé* for prostheses and orthoses.^42^ In this concept, the level of evidence and clinical meaningfulness of benefits provided for a medical device by a manufacturer is systematically tied to the level of possible coverage (maximum number of patients fitted per year) and reimbursement (payment amount). An additional requirement is that, whenever possible, French research sites should participate in a study, or the setting of a study should be transferable to the research and/or care setting in France. The benefit of the French model is that there is a cooperation between the HAS, clinical experts and the manufacturer in developing research projects and defining the relevant outcomes. If the study was conducted as agreed upon with the HAS, coverage is granted for a period of 5 years, after which it has to be renewed for another 5 years with another study that demonstrates that patients use the device in their daily lives. However, this coverage does not mean that the payment amount is fixed and secured, as discount negotiations may be initiated by the HAS on a yearly basis. Health-economic studies can support both the initial application and reimbursement negotiation and the 5-yearly renewals. Despite the benefits of being able to negotiate the study details with the HAS, the process is usually time-consuming and results in considerable delays in coverage compared to other European countries. In addition, the expectations of the HAS towards study design and outcomes are often so unique that manufacturers have to conduct specific studies that are of limited or even no value for coverage negotiations in other countries.

#### NORDIC COUNTRIES/SWEDEN

Sweden has a long tradition of a consensus-oriented culture that also applies to the healthcare system. As soon as a P&O product is available on the market, individual patient coverage may be claimed on a case-by-case basis. However, if general public coverage is pursued, especially for high-priced innovative products, a health-technology assessment is to be initiated by a health care professional (HCP).^43–45^ Manufacturers are not intended to do this as a clear need for the new treatment option must be requested by a HCP. This is the common approach to appraisals in 21 counties, with an evidence review being at the core of the assessment procedure. The diversity in additional assessment tools ranges from mini-HTAs to coverage with evidence development. The methodology required is the same for drugs, medical devices and P&O. It is comprehensible and achievable:^46^

- National health databases and registries^47,48^ must be used.
- Clinical Experts should be consulted for modeling.
- Health-economic studies required in cohorts < 65 years of age from a payer's and a social perspective.

One example for this is a cost-effectiveness study for Kenevo, an MPK for limited community ambulators, performed by Kuhlmann et al. It found ICERs of SEK 138,003 (€11,138/US$13,143) per quality-adjusted life year (QALY) gained for prosthesis users 65+ years with diabetes/vascular disease and SEK 114,772 (€9,263/US$10,930) per QALY gained for those with other etiologies.^49^

According to the published methodology,^46^ 3 levels of thresholds are used for appraisal: “low cost” with <SEK 100,000 (about US$ 10,000), “high cost” with >SEK 500,000 (about US$ 45,000) per QALY, and “very high cost” with >SEK 1,000,000 (about US$ 90,000) per QALY.

Health-economic studies may be seen as an essential aspect to obtain a public reimbursement policy in the counties.

#### POLAND

Currently microprocessor-controlled prosthetic technology is not covered by the public health care system. Nevertheless, due to the country's economic development and growth, its coverage is being discussed. There are two different pathways working in parallel to appraise health technologies including P&O that both take health economics into account to some extent. The pathway based on the new Act on Health Care Services Financed by Public Resources,^50^ introduced in 2012, is based on a well-described procedure of reimbursement decision-making for specific products with publication of the findings. The other one is based on the Reimbursement Act of 2011^51^ with an HTA process that always evaluates an entire class of products. Reimbursed products are then published in the list of medical devices dispensed to patients on professional prescription. During the HTA process, a “threshold price” is calculated^52,53^ that ensures that the ICER does not exceed three times the per-capita GDP (2019: US$ 15,595, resulting in a threshold of US$ 46,785).^54^

#### UNITED KINGDOM

The UK has 3 HTA bodies, each of which has its own approach to the assessment of health technologies for the National Health Service (NHS) trusts in England,^55^ Scotland,^56^ Wales^57^ and Northern Ireland^58^ (adapt National Institute for Clinical Excellence [NICE] guidance after confirming it is applicable locally), all of them mainly driven by health-economic evidence. Third-party assessments have to follow the HTA bodies' assessment methodology. The NICE has adopted a cost-effectiveness threshold of £20,000 (US$27,200) per QALY,^59^ which is a benchmark also used within the EU if no national thresholds exist.

The NHS England reviewed clinical outcomes as well as health-economic data for the clinical commissioning policy on microprocessor-controlled prosthetic knees in 2016.^60^ Though the majority of the available evidence was generated with C-Leg, the policy has granted coverage for MPKs in general under the condition that patients meet certain qualifying criteria.

Also in 2016, the National Institute for Health Research commissioned an HTA of the “*Orthotic Management of Instability of the Knee related to Neuromuscular and Central Nervous System Disorders including Economics*”,^8^ concluded that the evidence on the effectiveness of orthoses is limited, especially with regards to outcomes that are important to orthoses users, emphasizing the need for high-quality research on the effectiveness and cost-effectiveness of orthoses and the development of a core set of outcome measures.

As UK HTA reports are highly regarded by other European agencies and vice versa, these reports in the P&O segment in conjunction with the implementation of the Medical Device Directive must be taken seriously by manufacturers.

#### ASIA-PACIFIC

The implementation and development of health-technology assessments in the relatively new healthcare systems in Asian countries has taken place with a time delay compared to the USA and Europe. The growing necessity to provide the best value for the available resources while safeguarding accessibility to care has only recently led to an increasing number of Asian countries (Malaysia, Thailand, South Korea, Vietnam, Indonesia, Japan, and China) that have started implementing HTAs. However, the evaluation processes for clinical and economic evidence of healthcare technologies is often not yet formalized.^61^ In most cases, P&O is still treated as a separate sector that is not (yet) affected.

In general, the assessment of clinical outcomes is particularly complex since historically, many international pharmaceutical companies or medical device manufacturers have not conducted studies in Asia.

#### JAPAN

Japan achieved universal health insurance coverage in 1961.^61^ Healthcare spending as a share of GDP was 10.9%, the sixth highest among OECD countries, and per-capita expenses were US$ 4,519 in 2018.^62^ Today the country is ranked highly across numerous health indicators.^63^ After a 3-year HTA pilot testing phase, HTAs including health economic studies and budget-impact analyses were formally implemented in 2019.^62–64^

In general, a payer perspective is required in cost-effectiveness studies. In case the intervention has an impact on productivity, a social perspective according to the Core2health methodology^64,65^ is preferred. Japan has a policy of tight control of health care costs^64^ and is the first country using an algorithmic method for ICER-based pricing.^66^ That means that price reductions are executed in a 3-layer approach: 30% if the ICER is >5 million yen (about US$ 50,000) per QALY, 60% if the ICER is >7.5 million yen (about US$ 75,000) per QALY, and 90% if the ICER exceeds 10 million yen (about US$ 100,000) per QALY. For rare diseases, cancer or pediatric therapies, 50% higher thresholds are acceptable. Nevertheless, there is concern that this approach does not completely conform with ISPOR recommendations and does not use the ICER exclusively to demonstrate cost-effectiveness.^67^

Currently, P&O is not appraised for public reimbursement under this process. However, clinical outcome (effectiveness) is reviewed and published health-economic studies or public reports of HTA bodies are supportive to obtain public reimbursement. Coverage is subsequently determined by the 47 prefectures and 1,718 municipalities.^68^ Individual case-by-case reimbursement review is a common approach in P&O. If a policy for reimbursement is to be established, the opinion of a medical society and guidelines are required.

#### CHINA

In China a transformation process of the healthcare system is ongoing with the goal to achieve access of all citizens to the same healthcare standards in all provinces.^69^ The harmonization process for one national health-technology assessment standard is one part of it. The latest initiative was started in 2016 by the China National Health Development Research Center (CNHDRC) to develop HTA capacity and expertise by founding the China Health Policy and Technology Assessment Network which compromises 29 agencies, universities, hospitals and professional associations in 2016.^69–71^

An HTA guideline development process is also ongoing to strengthen the implementation of HTA, with a first publication of the China Guidelines for Pharmacoeconomic Evaluations in 2015 and a nationwide task force to continuously develop and refine them.^72^ The Chinese health insurance system is a patchwork of different types of mandatory and voluntary private health insurances. Most Chinese residents are covered by up to three types of basic social health insurance (BSHI), namely, Health Insurance for Urban Employees (HIUE), Health Insurance for Urban Residents (HIUR), and New Rural Cooperative Medical Scheme (NRCMS). This complex system has led to a diversity of multi-level payer plans.^69^ It has not been defined yet if health-economic studies will be used in the private health insurance sector. P&O is handled as a different sector, the role of HTA is not yet defined either.

Health-technology assessments of certain P&O technologies have been performed in a number of countries. **Table 1** gives an overview on the country, the technology assessed, the level of the HTA, and the institution that conducted or commissioned the HTA. In total, 16 HTAs were identified. Nine HTAs were conducted the payer's side, with 8 of them being reviews of published outcomes (level 1) and one being a CEA from the payer'sperspective. Two thirds of the HTA assessed a single product and 33% a class of products. HTA conducted by other parties included 6 analyses from a payer's perspective, thereof only one for a product class and two from the societal perspective, both for MPKs.

**Table 1:** Overview health technology assessment activities performed.

country	HTA level 1 outcome Review	HTA level 2 CEA payer perspective	HTA Level 3 CEA society perspective	Conducted by other parties A= association (industry) H= Health economic Institution M=Manufacturer R= Research group	Payer
**Canada**	C-Leg				P^6^
**Canada**		C-Leg			P^7^
**USA**	MP lower limb prostheses				P^5^
**USA**			MPK	A, H^10^	
**USA**		Genium		R^73^
**Germany**		C-Leg		H, M^40^
**France**	C-Leg				P HAS^74^
**France**	Kenevo				P HAS^75^
**France**	Rheo				P HAS^81^
**France**	Plié				P HAS^77^
**Italy**		C-Leg		R, M^78^	
**Italy**			MPK	R, M^79^	
**UK**	MPK				P^60^
**UK**	Orthoses neurologic knee instability				P^8^
**Sweden**		Kenevo		H, M, R^49^
**Sweden**		Osteointegration		R^80^
**Total publications**	8	6	2	7	9

## HEALTH ECONOMIC ANALYSES MAY HELP SUPPORT FUTURE COVERAGE OF TRUE INNOVATIONS

Health-economic analyses should primarily be performed for truly innovative products or those with high budget impact on the healthcare system to justify the efforts of the industry. This requires a well-developed research plan with scientific objectives that are relevant and beneficial to patients.^81^ As this is so important, it is desirable to have a transparent interaction process with payers whenever possible to obtain their perspective on what information is needed and which advancements are perceived valuable.

Several such evaluations may culminate in an accepted standard how to conceive a health-economic model that can guide smaller companies and payers in the future.

In the research & development process, health-economic analyses of manufacturers have to deal with the challenge of little or even no availability of objective data in the beginning and the need for several iterations and refinements with improving availability of data along the way. Therefore, the methodological approaches can be divided in four phases (**Table 2**). Further details and limitations (**Table 3**) have already been published by the authors elsewhere.^82^

**Table 2:** Health Economic Analyses in the R&D Process of manufacturers and in the product life cycle.^82^

Phase	Information based on HTA	Subsequent manufacturer's decisions
**Early-State Development**	Potential of a prosthesis/orthosis being cost-effective as part of manufacturer's investment decision prioritize between competing possibly cost-effective concepts or prostheses/orthoses; identify those parameters that have the largest impact on the likely cost-effectiveness of the prosthesis/orthosis in order to direct limited research funds.^5^	Design and management of prostheses and orthoses Regulatory and reimbursement strategy
**Mid-state development**	Feasibility check of cost-effectiveness based on first observational/small clinical studies Develop optimal clinical assumptions with clinical experts Develop a disease-specific model	Value proposition Health-economic study for payer review
**Late-state development**	Cost-effectiveness can be demonstrated Affordability is shown with a budget-impact analysis	Claims for reimbursement
**Product life cycle**	Re-calculate the health economic model with extended data Validate assumptions	Use to maintain or extend coverage

**Table 3:** Limitations to health-economic modeling and analyses in the development process and product life cycle.

	Limitation	Stage
**Implementati on**	Level of health economic expertise Resources (cost, time) Interdisciplinary cooperation (all relevant stakeholders)	Development process
**Intervention**	Innovation in medical devices is often a process of continuous incremental improvement. Short life cycle compared to drugs	Any stage
**Comparator**	Treatment standards are very often not established	Any stage
**Model inputs**	Manufacturer's access to national databases (cost, epidemiology) Resilient outcome measures	Product life cycle
**Decision**	Based on analyses that contain the best knowledge available at the time.	Any stage
**Optimum price setting**	Interaction of all stakeholders Patient potential (revenue) Estimated cost-effectiveness in daily practice	Early stage
**Investment**	Estimates per one health-economic study published by a manufacturer range from €50,000 to €100,000 (US$60,000 to US$120,000), depending on external and internal expert involvement and model input data processing	Product life cycle

## WILLINGNESS TO PAY AND SHIFT TO VALUE-BASED HEALTH-CARE FUNDING

An obvious important limitation of health-economic analyses is the ultimate determination of the “willingness to pay” of health-care payers (key figures for willingness to pay see **Table 4**). In addition, the willingness to pay varies between countries and sometimes even between different authorities or insurances within one country.^26–29^ Nevertheless, it is an important step towards meaningful healthcare research and a fact-based negotiation with payers. However, in times of restricted budgets, even well-performed health-economic studies do not automatically guarantee coverage and reimbursement of a product. For priority setting in health policies, a second aspect, affordability from a payer's perspective, must be reflected.^83^ This raises the challenge to the researching manufacturer to model the budget impact on resources consumed using national cohort data. If available, the effort to analyze the tremendous volume of data (DRG/ICD-10) is very time-consuming. So far, two publications for MPKs fulfill this concept in a German and Swedish context.^40,49^ However, despite demonstrating the affordability of an intervention, the decision to grant coverage and reimbursement is left to the payer. Shifting from a price-based to a value-based discussion may help promote this change and overcome the current barriers of capped reimbursement that attempt to force innovative technologies into existing billing schemes for established components, leaving little or even no room for the appropriate reimbursement of truly innovative components with proven clinical benefits. This will become increasingly important as patients are fitted in greater numbers with microprocessor-controlled components. In addition, HTA bodies and payers should not only demand high-quality research from manufacturers of innovative devices but also value it by setting the same bar for technology followers, rather than letting them get away with just claiming equivalence to predicate devices.

**Table 4:** Willingness to pay assessment key figures.

Cost-effectiveness	Budget Impact
Defined threshold ^46,53,55,59,61^	Absolute costs and savings^83^
Country's per-capita gross domestic product (GDP)^84^	
ICER accepted for payment as reference US dialysis USD 50,000^85^	

Finally, because health-economic modeling and analyses are currently uncommon in R&D processes, prosthetic manufacturers are advised to benefit from the experience of pharmaceutical and implantable medical device companies and health economic research institutions to shorten the learning curve and minimize waste of investments.

## LIKELY CONSEQUENCES OF THE ADOPTION OF HEALTH-ECONOMIC EVALUATIONS ON THE MARKETS FOR PROSTHETIC AND ORTHOTICS

The adoption of health-economic evaluations in P&O would require a substantial expansion of clinical research capabilities, staff and funding among manufacturers. Given the current structure of the prosthetic manufacturer community with its many smaller businesses, this will present a substantial challenge. It may result in a further partition of the manufacturer community, the need to collaborate and cooperate, and perhaps even mergers. Smaller companies that are unable to absorb the additional investments will likely have to focus on me-too products that fit into the limitations and restrictions of the current reimbursement systems.

However, even the group of bigger manufacturers will likely have to make some tough decisions. Clinical and health economic research is expensive and can therefore only be done for new high-price innovative products. Thus, manufacturers will have to decide which of their products and R&D projects they want to support with this additional investment, leaving some of their products or even entire product categories vulnerable.

Another challenge that may arise is that some products may deliver their biggest benefits with a good incremental cost-effectiveness ratio to a relatively small group of patients that may not be big enough to justify the R&D and research expenses. The phenomenon of disappearing innovation in small markets is well known from the pharmaceutical industry. To prevent that from happening, there are proposals to develop tests to identify likely responders and reimburse the treatment of these patients at higher rates to justify the R&D expenses and keep these small markets economically attractive.^86^ In P&O, the procedure of trial fittings could serve that purpose.

Another challenge to innovative manufacturers is the burden to beat the path for new technologies. Followers wait until a favorable reimbursement infrastructure has been established, and then launch me-too products that do not require the same level of evidence as the predicate device, if any. As these manufacturers save the substantial expenses for clinical and health-economic research, they are usually able to offer their devices at lower prices. This creates a competitive advantage for me-too manufacturers in many markets and makes it more difficult for innovative companies to recoup their R&D investments.

Therefore, increasing requirements for demonstrating clinically meaningful patient outcomes and health-economic evidence may be perceived as a short-term advantage but long-term disadvantage for manufacturers that are willing to make the necessary research investments. However, a positive development can meanwhile be observed in the European Union that now regulates product entrance in the market by requiring clinical data generated for every individual product and continuous post-marketing patient safety monitoring. This precludes the manufacturers of me-too devices from simply claiming equivalence with predicate devices. A similar model would be desirable for the US and Canada to maintain the fiscal incentives of research-supported innovation. This would be in the best interest of patients, suppliers, providers, and health care payers, as the vast majority of prosthetic and orthotic devices are currently not strictly regulated by research requirements for safety and effectiveness set by regulatory bodies.

## CALL TO ACTION

Manufacturers of prosthetic and orthotic products need to recognize the increasing prevalence and importance of HTAs for medical device coverage and reimbursement. The requirements to the evidence to be demonstrated are currently only beginning to surface. Unlike the past, when most manufacturers waited for an industry leader to come forward and do the work for their product and then claimed, without any proof, that these studies also applied to their products, all manufacturers are called upon to contribute to the body of evidence for certain product categories, such as MPK or microprocessor-controlled feet. That would both substantially enlarge the body of evidence for the respective product category and fix the current limitation that study results for one product are assumed to apply to the entire category without any proof. Evidence that is more representative of the product diversity in the market would certainly be much more compelling to payers. In that context, industry and professional associations are called upon to support this process of evidence generation by commissioning independently conducted systematic reviews of the existing literature.

Finally, health insurances and payers for O&P products in general are called upon to no longer reduce health economy in this field to simple cost savings but apply value-based approaches that are similar to those already used for other medical devices but reflect the peculiarities of O&P. Ultimately, considerable transparency between payers and their willingness-to-pay thresholds for valued clinical benefits will enhance the willingness of manufactures to pursue continued innovation with a sustainable cost model. As the emphasis on demonstrated clinical effectiveness and value increases, policy makers are advised to hold individual components to comparable standards of demonstrated performance to ensure that the costs of development in this value-based model are born equally across all component developers and manufacturers. Nevertheless, health economy is unable to answer the question how the inevitably incremental cost to pay for innovative, yet cost-effective services and products shall be funded. Societies will need to have an open discussion to find a compromise between stimulation of medical innovation and affordability of the healthcare systems.

## DECLARATION OF CONFLICTING INTERESTS

Both authors are full-time employees of Otto Bock Healthcare.

## SOURCES OF SUPPORT

Ottobock allowed both authors to work on this article during their regular working hours and use the company's office equipment (computers, Internet access, etc.).

## AUTHOR SCIENTIFIC BIOGRAPHY

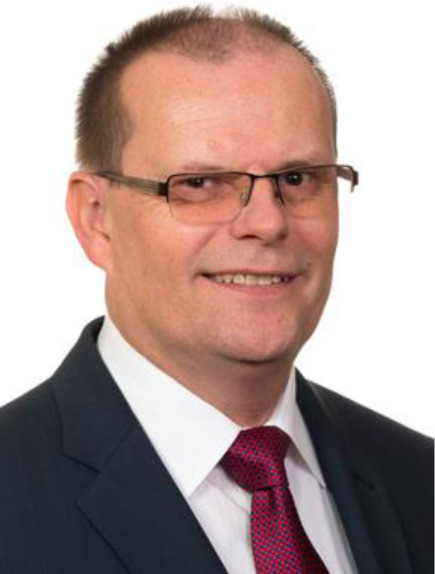



**Andreas Kannenberg** graduated from Charité Medical School at Humboldt University in Berlin, where he also wrote his dissertation in exercise physiology. He had worked as a physician in Germany for more than 10 years before joining Ottobock in 2003. He has been serving as the Executive Medical Director North America since 2013. He coordinates Ottobock's clinical research in the Americas and is part of its global medical and research team. In his prosthetic and orthotic research, he has been focusing on microprocessor-controlled prosthetic knees, microprocessor stance and swing control orthoses, and multi-articulating hands. A recent new focus of his research has been the impact of prosthetic components on musculoskeletal pain in individuals with lower-limb amputations. He is an affiliate member of the American Academy of Orthotists and Prosthetists (AAOP) and a member of the Clinical Education Committee of the American Orthotic and Prosthetic Association (AOPA).

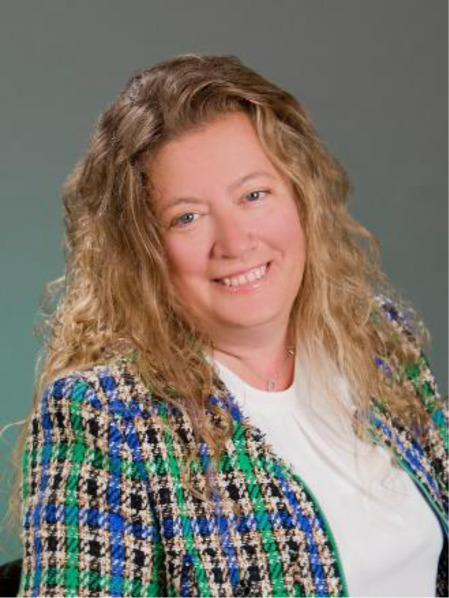



**Susanne Seidinger** graduated as a Doctor of Veterinary Medicine from the Veterinary University of Vienna, Austria, and has gained significant experience in medical affairs, health economy, healthcare policies, market access and reimbursement roles in the medical device and pharmaceutical industry. In addition, she acquired a certification in Health Technology Assessment from Sheffield University. She joined Ottobock in 2017 and has been serving as Director Global Market Access Management since 2019. The focus of her research has been the entire spectrum of health economic studies in highly innovative medical, prosthetic and orthotic products.
